# An Investigation of Several Physicochemical Characteristics, as Well as the Cholesterol and Fatty Acid Profile of Ice Cream Samples Containing Oleogel, Various Stabilizers, and Emulsifiers

**DOI:** 10.3390/gels9070543

**Published:** 2023-07-04

**Authors:** Cihat Ozdemir

**Affiliations:** Food Science Department, Oltu Vocational School, Atatürk University, Erzurum 25400, Turkey; cozdemir@atauni.edu.tr

**Keywords:** ice cream, beeswax, organogelators, microscopic appearance, color, fat destabilization, cholesterol, fatty acid, Palsgaard, konjac gum

## Abstract

This study investigated the possible usage of beeswax oleogels instead of milk fat as a fat source in ice cream production and konjac gum as a stabilizer instead of salep. For this aim, 12 different ice cream samples were prepared using various fat and oil sources (milk fat and oleogel), stabilizers (salep and konjac gum), and emulsifiers (monoglyceride (MG), Palsgaard (PG), and no emulsifier/emulsifier-free (NE)). It was determined that the overrun ratio of ice cream samples containing milk fat was higher than that of samples containing oleogel, and the viscosity of the ice cream mix containing Palsgaard and oleogel was greater than that of the mix with other treatments (milk fat, MG, and NE). While the first dripping time of the samples with PG and konjac gum was longer compared to the samples without emulsifier (NE) or monoglyceride (MG), the complete melting times of the samples were close to each other. Whereas the dissolution rate of the samples with salep was higher than that of the samples with konjac gum, the fat destabilization ratios of the samples with oleogel and konjac gum were lower than those of the samples with milk fat and salep. The fat destabilization ratio of samples containing PG as an emulsifier was statistically higher (*p* < 0.01) than that of samples containing MG and NE. It was found that the unsaturated fatty acid (C18:1, C18:2, and C18:3) content of the samples containing oleogel was significantly higher (*p* < 0.01) than that of the samples containing milk fat. However, butyric and caproic acids, which are aliphatic fatty acids, were found to be deficient in the samples to which oleogel was added instead of milk fat. As to the microscopic appearance, while water and oil particles were not homogeneously dispersed in the ice cream samples with oleogel, they were fully homogeneously dispersed in the milk fat-added ice cream samples. In addition, it was determined that panelists preferred the samples with added milk fat as fat source, salep, and PG as emulsifier. Among the samples with added oleogel as the oil source, they liked the sample added with oleogel as fat source, konjac gum, and no emulgator more.

## 1. Introduction

Ice cream is a complex food with several structural components. Ice cream is a frozen dairy product that contains fat, sugar, non-fat milk solids, flavors, emulsifiers, and stabilizers. Ice cream is a fat-rich, delicious dairy product that is enjoyed by many people all over the world. Since ice cream contains a high percentage of milk fat (9%), consumers are more likely to develop obesity and cardiovascular disease. The production of medium-fat, low-fat, and non-fat ice cream reduces the risk of obesity and cardiovascular disease due to a lower intake of saturated fats [1]. Fat reduction has a negative impact on the texture of the ice cream because fat is an important component of ice cream ingredients and contributes significantly to the internal structure of the ice cream as well as its creaminess and smoothness [2].

Some manufacturers want to reduce milk fat and use vegetable oils in ice cream formulation; therefore, a significant portion of ice cream can be made with naturally saturated oils such as palm kernel oil, palm oil, or coconut oil, which are commonly used for this purpose [3]. Trans-fatty acid intake is caused by the consumption of hydrogenated oils. It is implied that lowering saturated fat and eliminating trans-fat lowers the risk of cardiovascular disease and other diet-related disorders [4]. Ice cream manufacturers would be very interested in reduced saturated fat and the ability to use domestic oils in ice cream formulation. Coconut and palm kernel oils have traditionally been used in the manufacture of ice cream. While coconut oil has a high ratio of saturated fatty acids, up to 92%, palm kernel oil has a high ratio of saturated fatty acids, up to 80%. The internal structures of ice creams containing oils with high saturated fatty acids have been determined to be of higher quality [5].

Solid fat plays an important role in maintaining the structure of ice cream, and a small amount of crystalline fat is required to maintain the instability that allows the structure to develop; therefore, solid fat in ice cream cannot be replaced with liquid oil. However, because saturated fat is linked to an increased risk of heart disease, the WHO recommends that saturated fat intake be constrained to no more than 10% of daily energy intake [6]. The use of large volumes of liquid edible oils with low oleogelator concentrations has sparked a great deal of interest as a way to reduce the use of saturated fats [7]. Oleogelators compress liquid oils into a hard material without changing their fatty acid composition; thus, liquid oils in oleogel form can be used to reduce saturated fat in ice cream formulation [8]. The crystallizing properties of food-grade waxes such as beeswax (BW), candelilla wax (CDW), carnauba wax (CBW), and rice bran wax (RBW) as oleogelators may play an important role in the structure of ice cream. Among them, RBW demonstrates a superior ability to structure oil [9]. However, the type of oleogelators and the methods for applying oleogels to foods are still quite limited. Since ice cream manufacturers need solid fat, such as milk fat, coconut oil, or palm oils, oleogels allow them to employ a considerably higher concentration of unsaturated fatty acids [10]. As a result, the use of saturated fat can be reduced. Some food-grade oleogelators have been proposed recently. Beeswax oleogelation can benefit the production of ice cream [8]. This would allow the use of more liquid oil as a fat source with high levels of polyunsaturated fatty acids. It is critical that the amount of non-dairy fats used in ice cream be limited. Rice bran wax (RBW) oleogel (90% oleic sunflower oil + 10% RBW) was used in a study to replace solid fat in ice cream, which was produced using different emulsifiers (mono and diglycerides, as well as polysorbate 80). It was discovered that the overrun of ice cream samples containing oleogel was greater and the air bubbles were smaller compared to the control, but the fat destabilization and meltdown ratios were comparable [11].

Stabilizers are used in ice cream production to increase the viscosity of the mix, prevent the formation of ice crystals during processing and storage, and preserve its structure by slowing melting during the consumption stage. In Turkey, salep is commonly used as a stabilizer in the production of ice cream. The use of konjac gum powder as a stabilizer in ice cream was also developed not only to investigate ice cream quality but also to reduce ingredient costs. Konjac gum is considered safe (GRAS) and contains D-glucose and D-mannose. It is a neutral polysaccharide and has unique properties such as thickening, gelling, texturizing, and water binding. The viscosity of ice cream prepared with 0.3% konjac gum was higher than that of the other treatments (0.1% and 0.5% konjac gum adding) [12]. Xanthan gum (XG) has been found to improve stability during freezing and the oil immersion of egg white protein [13]. It has been concluded that adding a mixture of konjac gum, sodium carboxymethyl cellulose, carob bean gum, and carrageenan as a stabilizer in Kahramanmaraş-type ice cream production could be an alternative to the use of salep [14].

Wu et al. evaluated meltdown behavior by varying levels of stabilizers (polysorbate 80). Additionally, Wu et al. determined in their study that the fat destabilization value of ice cream samples changed from 8.8 to 73.2%, and the mix viscosity ranged between 0.0220 and 0.2904 Pa.s. Ice cream viscosity and fat destabilization increased as stabilizer levels increased [15]. Emulsifiers promote the adsorption of small-molecule surfactant at the oil/water interface and makes fat destabilization more likely during the freezing process [16]. Monoglycerides (MG) are a common emulsifier in ice cream formulations. According to some studies, MG enhanced the structure and meltdown stability of ice cream [17]. Subsequent tests of overrun, melting properties, and acidity all yielded highly significant results. In addition, significant results for viscosity were obtained in relation to treatments and storage period. The difference in stabilizer/emulsifier combination had a significant impact on body/texture, flavor, and taste [18]. Palsgaard (PG) is an emulsifier and stabilizer used in ice cream. PG also assists in the development of new products and the optimization of existing formulations. A study investigated the effects of PG on the stabilization and emulsion of ice creams with reduced saturated fat levels. They found that the sensory properties of low-fat ice cream samples containing Palsgaard were comparable to the control sample [19]. 

The goal of this study was to look into the potential use of beeswax oleogels in ice cream. When the amount of saturated fat in the ice cream is reduced, it becomes softer. We aimed to show that various emulsifier and stabilizer systems can be used to produce ice cream with the desired texture and edible qualities. The other goal of this study is to produce alternative ice creams that are less expensive than other methods but are completely liquid oil-based and do not contain trans-fat. They were then tested to see if this structured ice cream production method was suitable for forming the desired texture and consistency in ice cream. 

Since ice cream typically has a high fat content (10–14%), preparing low-fat diet versions by replacing milk fat with oleogel will help make ice cream healthier by reducing the amount of fat. Ice cream production technology aims to produce alternative and innovative ice cream at a lower cost than other methods. Since salep has been used as a stabilizer for a long time in Turkey, its amount is gradually decreasing. For this reason, konjac gum will be an important alternative in place of salep as a stabilizer in ice cream production.

## 2. Results and Discussion

### 2.1. Physicochemical Properties of Milk, Cream, Sunflower Oil, and Oleogel 

Some physicochemical properties of milk, cream, sunflower oil, and oleogel used in ice cream making are given in Table 1.

### 2.2. The Physicochemical Analysis of the Mix and Ice Cream Samples

The results of physicochemical analysis of the mix and ice cream samples are given in Table 2. 

**Figure 1:**
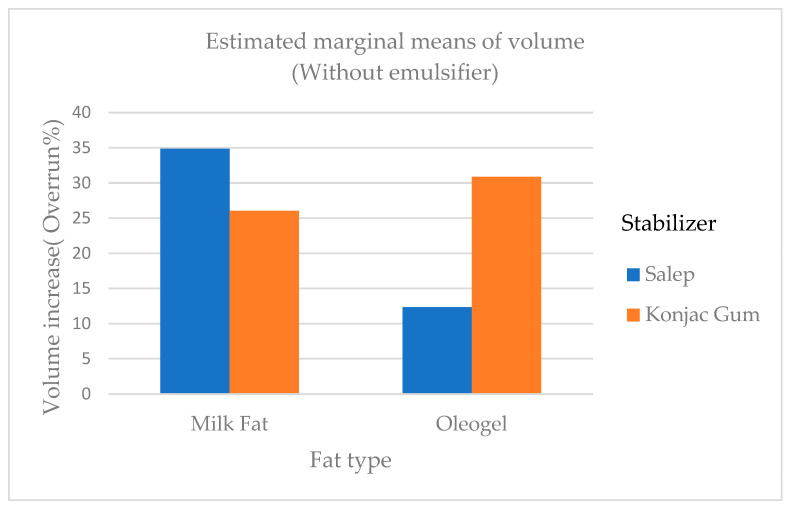
The overrun ratios of ice cream samples added cream, oleogel, stabilizers and emulsifiers.

The dry matter and ash ratios of ice cream samples ranged from 29.82 to 33.13% and 0.72 to 0.97%, respectively. The milk fat ratio of the mixed samples ranged between 4.65 and 5.10%. Sulejmani and Demiri [20] made the ice cream samples with stevia and emulsifiers and found that dry matter, ash, and fat ratios ranged between 36.12 and 56.62%, 0.34 and 0.63% and 0.00 and 3.96%, respectively. The oil ratios ranged between 6.15 and 6.50. The oil ratio of samples with added oleogel was statistically higher (*p* < 0.01) than that of milk fat. The dry matter ratios noted by Sulejmani and Demiri [20] were higher than those of the samples in this study, but the ash and fat ratios were lower. Consistent with the results of the researchers (0.16% and 0.22%), the acidity of the samples varied between 0.13% and 0.18%. The pH of the samples ranged from 5.91 to 6.71. The results of the same research found that the pH of the ice cream samples was between 5.92 and 6.48, consistent with this study [20]. The overrun ratios of the samples ranged from 6.50 to 42.10%. It was found that the overrun ratio of ice cream samples containing milk fat was greater than that of samples containing oleogel (Figure 1). In a study, ice cream was made by replacing 50% and 100% of its milk fat with sunflower oil. It was discovered that it had the lowest overrun percentage and melt resistance among all ice creams and our results were consistent with the findings of this research [21].

**Figure 2:**
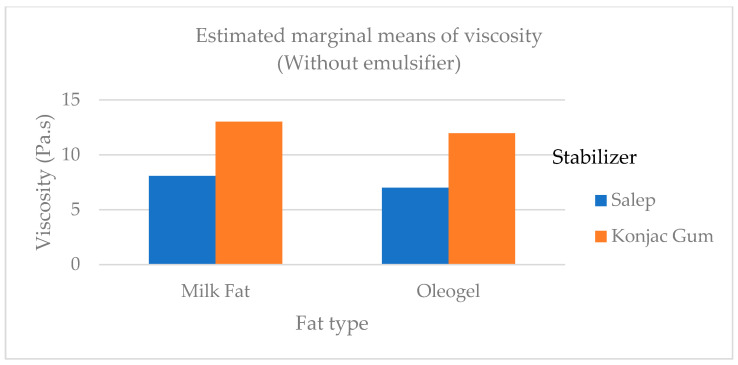
The viscosity of the ice cream mix added cream, oleogel, and stabilizer.

Corradini and others found that when palm oil and coconut fat were added to the ice cream mix, the overrun of ice cream samples ranged between 48.8 and 50.4% [5]. The findings of Corradini et al. [5] were higher than the findings of this study. Viscosity is one of the most important properties of an ice cream mix as it frequently contributes to the desirability of ice cream’s body and texture. At 20 rpm, the viscosity of the mix samples ranged from 10.06 Pa.s to 28.00 Pa.s. At 50 rpm, the viscosity ranged from 7.00 to 14.70 Pa.s. The viscosity of ice cream mixes containing fat and conjac gum were generally higher than that of mixes containing other treatments (Figure 2). In another study, Mariano and Alamprese [22] added gelator at two different levels to the ice cream mix and found that the mix viscosity of the samples with added gelator was statistically lower (*p* < 0.01) than that of of the samples with milk fat, unlike the findings of this study. In a study by Ozdemir et al. [23], they found that at a sliding speed of 20 rpm, the viscosity of ice cream samples ranged between 7.03 and 9.28 Pa.s. This study’s viscosity results were higher than those of the study by Ozdemir et al. [23]. According to Minhas et al., the viscosity of ice cream samples ranged from 0.29 to 1.17 Pa.s. [24]. In this study, Minhas et al. [24] reported viscosity values in ice cream that were lower than our findings. This kind of situation can be caused by various ice cream mix compositions as well as various stabilizers and emulsifiers.

### 2.3. The Melting Properties and Fat Destabilization of Ice Cream Samples

The melting properties and fat destabilization of ice cream samples are given in Table 3.

**Figure 3:**
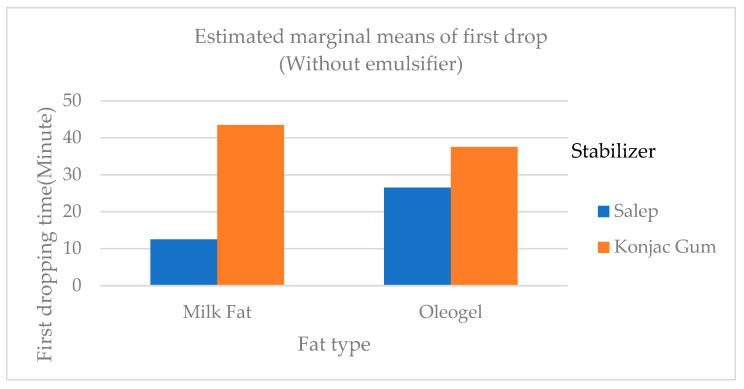
The variation of the first dropping times of samples of ice cream containing cream, oleogel, stabilizer, and emulsifier.

In general, the first dropping and complete melting times of konjac gum ice cream samples were statistically longer (*p* < 0.01) than those of salep-added samples (Figure 3). Although the first dropping times of samples containing PG (Palsgaard) (samples C and I) as an emulsifier with konjac gum as a stabilizer were longer than those of samples containing no emulsifier and MG (monoglyceride), the total melting times of the samples were similar (Table 3). The melting ratio of the samples containing salep (Samples A, B, C, G, H, and I) was generally greater than that of the samples containing konjac gum (Table 3). The 0.7% konjac gum addition resulted in the highest melting score when compared to the other konjac gum additions (0.1, 0.3, and 0.5%) and control (commercial stabilizer). This could be attributed to the high fiber content of konjac gum being associated with increased viscosity and water binding ability in ice cream [16]. The findings of this study were similar to those of Metwaly et al. [18].

Guven et al. [25] produced Kahramanmaraş-style ice cream using only salep, locust bean gum (LBG), CMC, guar gum, and S. alginate combinations and discovered that samples containing only salep dropped the earliest, which corresponded to the findings of this study. Another study looked into the possibility of using konjac gum as an alternative to salep, which is commonly used in ice cream production. As a result, it was determined that it can be used alone or in combination with salep to make ice cream. The melting time of samples containing salep was shorter than that of samples containing other stabilizer combinations [26]. The G sample (salep as a stabilizer, milk fat without emulsifier) took the longest time to completely melt.

**Figure 4:**
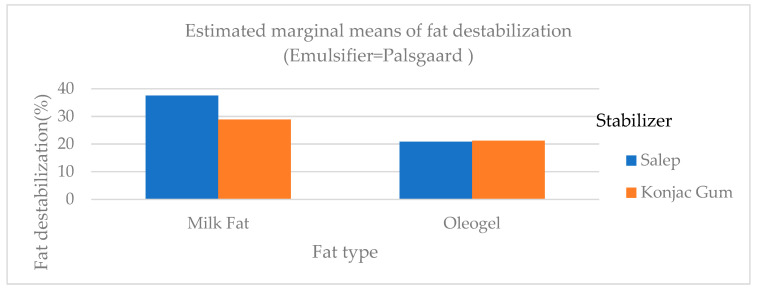
The difference in fat destabilization of ice cream samples that included milk fat, oleogel, and PG.

Fat destabilization ratios of ice cream samples ranged from 18.71 to 37.50% (Table 3). The fat destabilization ratios of samples containing oleogel and konjac gum were lower than those of samples containing milk fat and salep (Figure 4). The fat destabilization ratio of samples with PG as an emulsifier (C, F, I, and L samples) was statistically higher (*p* < 0.01) than those of samples with MG and samples with no emulsifier (NE). Amador et al. [27] made ice cream using an emulsifier at different ratios and found that fat destabilization values were between 6.6% and 36.0%, consistent with the findings of this study. In another study, Ozdemir et al. [23] discovered that the fat destabilization ratios of ice cream samples with different sweeteners ranged between 14.62 and 42.40%, which was consistent with the findings of this study.

### 2.4. The Color Values of Ice Cream Samples

Table 4 illustrates the color values of ice cream samples.

**Figure 5:**
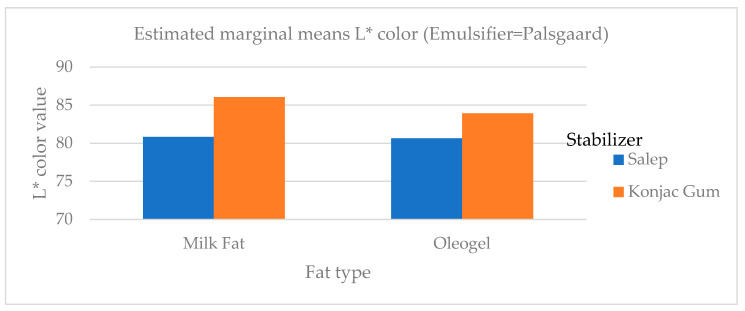
The variation in L* color values of ice cream samples that included cream and oleogel.

While the L value expresses brightness in daylight (black and white), the a* value represents green-red color, and the b* value represents blue-yellow. Consumers mostly prefer white in plain ice cream. The L* color scores, which indicate the white color level of ice cream samples, ranged from 80.66 to 87.15. The addition of oleogel to the ice cream mix did not affect the L* color value of the ice cream mix. The differences in L* values between samples with different emulsifiers were not statistically significant (*p* < 0.01). However, the addition of oleogel and salep reduced the L* color value (Figure 5). In research by Ozdemir et al. [23], it was found that the whiteness value (L*) of the ice cream samples ranged from 82.02 to 85.51, which was consistent with this study. In another study, Corradini et al. [5] discovered that the L* color value for ice cream samples containing palm oil and coconut fat ranged between 57.7 and 64.5 [5] and detected lower L* color values than this study.

### 2.5. The Fatty Acid Composition of Ice Cream Samples

Table 5 shows the fatty acid composition of ice cream samples.

**Figure 6:**
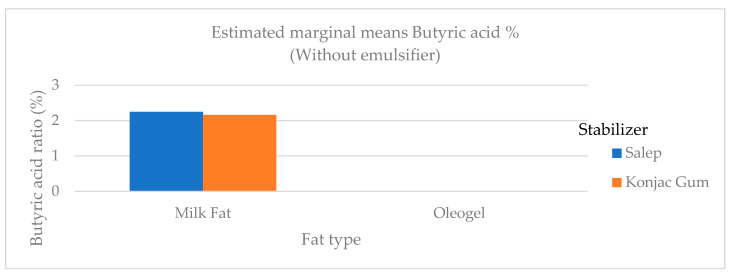
The variation in the butyric acid ratio of ice cream samples that included cream and oleogel.

**Figure 7:**
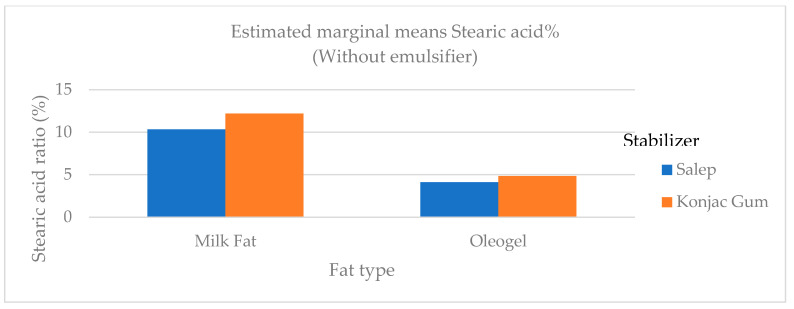
The variation in the stearic acid ratio of ice cream samples that included cream and oleogel.

**Figure 8:**
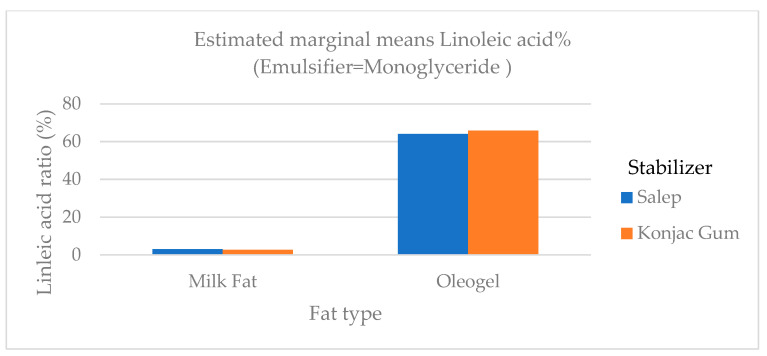
The variation in the linoleic acid ratio of ice cream samples that included cream and oleogel.

**Figure 9:**
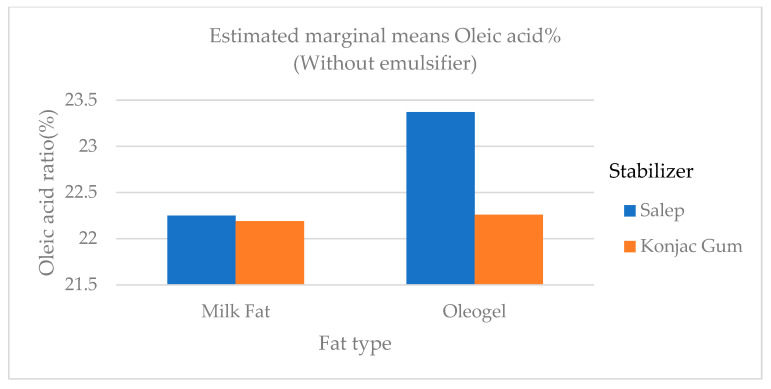
The variation in the oleic acid ratio of ice cream samples that included cream and oleogel.

Quantities of butyric and caproic fatty acids, which are low-chain fatty acids, were higher in ice cream samples containing milk fat than in samples containing oleogel (Figure 6). Butyric and caproic acids are base fatty acids found in milk fat. The low-chain fatty acids were barely detectable. In ice cream samples containing milk fat, the amount of long-chain saturated fatty acids (C14:0, C16:0, and C18:0), which would not be preferred in terms of nutritional aspect, was significantly higher (*p* < 0.01) than in samples containing oleogel (Figure 6). As stated in the studies, a reduction in saturated fat in human nutrition lowers the risk of cardiovascular disease and other diet-related disorders [4]. The level of polyunsaturated fatty acid (C18:2 and C18:3) in samples containing oleogel was significantly higher (*p* < 0.01) than in samples containing milk fat (Table 5 and Figure 7, Figure 8 and Figure 9). Nazarewich et al. [28] made oleogel from tomato seed oil and found that the tomato seed oil added to ice cream increased the unsaturated fatty acid amount, consistent with this study. However, the samples containing oleogel instead of milk fat did not contain aliphatic fatty acids or butyric and caproic acids. This can affect negatively the taste of ice cream samples with added oleogel (Table 5). In one study, Corradini et al. [5] found that adding palm oil and coconut fat to the ice cream mix did not affect the fatty acid profile. However, this study discovered that oleogel prepared with sunflower oil significantly (*p* < 0.01) altered the fatty acid profile (Table 5).

### 2.6. The Microscopic Appearance of the Samples of Ice Cream

**Figure 10:**
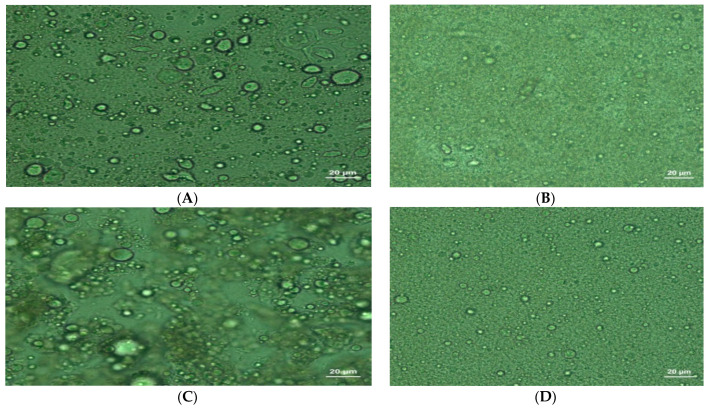
Microscopic images of the ice cream samples. (**A**) The microscopic appearance of samples containing salep and oleogel. (**B**) The microscopic appearance of samples of salep- and milk fat-added ice cream. (**C**) The microscopic appearance of samples of oleogel-, salep-, and Palsgaard-added ice cream. (**D**) The microscopic appearance of samples of konjac gum- and milk fat-added ice cream.

The microscopic appearance of the samples of ice cream is given in Figure 10.

In the microscopic appearance of samples of oleogel-added ice cream, while the oil and water particles were not dispersed as homogeneously (Figure 10A,C) as in the samples of milk fat-added ice cream, components were dispersed very homogenously (Figure 10B,D). The fat destabilization ratios of the samples of oleogel-added ice cream were lower than those of the samples containing milk fat (Figure 4), consistent with their microscopic appearance.

From the correlation test results (Table 6), there is a negative correlation between overrun and complete melting time (r = 0.7, *p* < 0.01). The complete melting time of the samples with high volume increase was shortened. Ice creams with low volume increase melt faster. It was determined that slower melting in ice cream samples was due to lower air transfer due to the large volume of air, but the main reason was low fat stabilization [29]. There is a negative correlation between the overrun and fat ratio (milk fat/oleogel) (r = 0.8, *p* < 0.01). As the fat ratio increased, the volume decreased. However, Güven et al. [30] found that low-fat ice creams had low volume. In ice cream samples, as the ratio of saturated fatty acids (C:4, C:6, C:14, C:16) increased, the volume rate increased (positive correlation) as well, and as the unsaturated fat ratio increased, the volume decreased. It is noted in Table 5 that the volume increase of ice cream mix with added oleogels, which are rich in unsaturated fatty acids, was lower than that of milk fat ice cream. There is a positive correlation between the amount of milk fat or solidified oil and complete melting time (r = 0.7, *p* < 0.01). As the amount of fat increased, the completed melting time also increased (Table 2 and Table 3). The fat destabilization decreased as the fat ratio increased in the ice cream samples. If the emulsifier and stabilizer are insufficient, in ice cream, all the fat or oil can not be linked to the mix. Hatipoglu [31] found that as the fat content in ice cream increased, the volume rate also increased, but the viscosity of the ice cream mix samples decreased.

There was a positive correlation between pH level and fat destabilization (r = 0.55, *p* < 0.01). As the pH level increased, the fat destabilization level also increased. This suggests that it is important to have a higher pH value for the fat destabilization value to be high in ice cream production. In ice cream samples with a high ratio of saturated fatty acids (C: 4, C:6, C:14, C:16), the complete melting time decreased (negative correlation), but as the ratio of unsaturated fatty acids increased, complete melting time decreased (*p* < 0.01). In Table 3, it can be seen that the complete melting times of the samples with added oleogel (G, H, F, J, K, L) are significantly (*p* < 0.01) higher than those of the samples with added milk fat as parallel correlation results. The first dripping time was positively correlated with viscosity results (r = 0.81, *p* < 0.01), but the differences between viscosity results did not affect the full melting time (Table 6 and Table 7).

### 2.7. Separation of Ice Cream Samples Using PCA (Principal Component Analysis)

**Figure 11:**
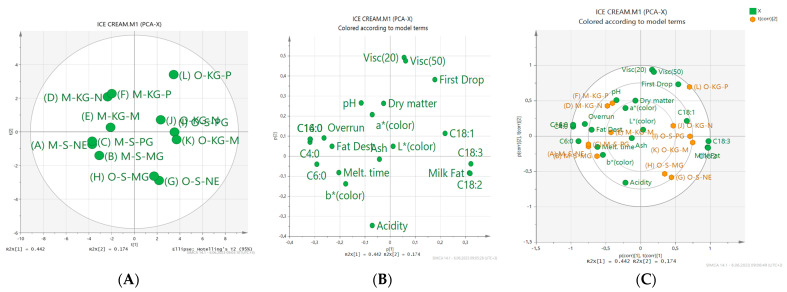
Score scatter plot (**A**), loading scatter plot (**B**), and biplot (**C**) of PCA analysis (PC1 versus PC2) for the components of the ice cream.

The separation of ice cream samples using PCA are given in Figure 11A–C.

PCA was applied to show the differences in the chemical, physical, and fatty acid composition of the samples. The effect of the first two main components, PC1 and PC2, on the total variation was determined as 61.6%. Samples were collected in 4 regions: the D, E, and F samples were concentrated in the 1st region, the A, B, and C samples in the 2nd region, the I, J, and L samples in the 3rd region, and the G, H, and K samples in the 4th region.

In Figure 11B, the dry matter, ash, acidity, pH, overrun, fat destabilization, melting time, a color, b color, C4:0, C6:0, C14:0, and C16:0 are on the left. Milk fat or oleogel ratios, first drop, L color, viscosity (20 rpm and 50 rpm), and fatty acids (C18:0, C18:1, C18:2, and C18:3) are on the right. When Figure 11C is examined, it can be seen that the highest dry matter ratio values were found in the F sample, the highest amount of oil in the H sample, the highest ash content in the J sample, and the highest pH value in the A sample. Moreover, it was found that the highest acidity ratio (%) was in the G sample, the highest overrun in the B sample, and the highest viscosity (20 rpm and 50 rpm) values in the L sample. Again, the highest values in the L sample were the first drop time, in the G sample the complete melting time, and in A sample the melting ratio was determined. Among the color values, the highest L* color value was in the K sample, a* color value in the L sample, and the highest b color value was detected in the A sample. 

When the fatty acids values are investigated in the same figure, the highest values, namely C4:0 in the A and B samples, C6:0 in the A sample, C14:0 and C16:0 in the E sample, C18:0 in the B sample, C18:1 in the I sample, C18:2 in the K sample and also in the I and L samples C18:3, were found.

### 2.8. Results of Sensory Analysis of Ice Cream Samples

C samples with added milk fat, salep (stabilizer), and Palsgaard (emulsifier) were preferred by panelists. Panelists scored the G sample with salep and oleogel lower than the I sample with salep, Palsgaard, and oleogel.

## 3. Conclusions

In the results of this study, it was determined that konjac gum could be used instead of salep to make ice cream. The overrun of the mix was higher when oleogel was added to the ice cream than in the samples with added milk fat. Panelists gave lower ratings to samples containing oleogel. However, the level of polyunsaturated fatty acid (C18:2, and C18:3) in oleogel-added samples was higher than in milk fat-added samples. Butyric and caproic acids, which are aliphatic fatty acids, were not present in the samples containing oleogel instead of milk fat. The situation can have a negative impact on the organoleptic qualities of ice cream samples that have been produced with oleogel. When oleogel is used as a fat source, ice creams can be flavored with low-chain fatty acids such as acetic and butyric acids. Fat destabilization of ice creams made using Palsgaard as an emulsifier was higher than ice creams made without an emulsifier or by adding monoglyceride as an emulsifier. The use of oleogel as a fat source in ice cream production significantly reduced the amount of saturated and long-chain fatty acids (C14:0, C16:0, and C18:0) in ice cream. The panelists preferred the samples added milk fat as a fat source, salep as stabilizer, and PG as an emulsifier. 

## 4. Materials and Methods

### 4.1. Material

Milk, cream, and sunflower oil were supplied by local markets in Erzurum, Turkey. Sugar (Konya Sugar Trade and Industry Co., Konya, Turkey) was supplied by local markets. Salep (flour made from the tubers of the orchid genera), Palsgaard, monoglyceride, and konjac gum (KG) were supplied by commercial markets, and skimmed milk powder was supplied by the Pınar Dairy Products Co. (Istanbul, Turkey). Finally, beeswax (BW) was purchased from KahlWax (Kahl GmbH & Co., Trittau, Germany).

### 4.2. Methods

#### 4.2.1. Beeswax Oleogel Preparation

To prepare the oleogel, beeswax was added to refined sunflower oil in a stainless steel container at 5 wt percent. The temperature of the mixture was raised to 80 °C to melt the wax. In order to guarantee a full and homogeneous distribution of the wax in the oil, the hot mixture was vigorously homogenized at 80 °C at 10.000 rpm for 5 min using a homogenizer (Ultra-Turrax^®^ T 25; IKA, Staufen, Germany) equipped with a dispersing tool (S25 N—18 G—ST-stainless steel). The samples were then ready for incorporation into the ice cream mix [32].

#### 4.2.2. Ice Cream Manufacture and Sampling

Ice creams were prepared in the laboratory of Atatürk University Faculty of Agriculture. In this study, twelve (12) different ice cream samples were produced using various fat and oil sources (milk fat and oleogel), stabilizers (salep and konjac gum), and emulsifiers [monoglyceride (MG), Palsgaard (PG), and none emulsifier (NE)]. Ice cream samples were made with 1500 g milk (3% milk fat or non-fat), 40 g non-fat milk powder, 90 g raw cream (50% milk fat) or oleogel, 300 g sucrose, 15 g stabilizers salep or konjac gum (KG) and 8 g emulsifiers’ monoglyceride (MG) or Palsgaard (PG). At A, D, G, and J samples, emulsifiers were not used (NE) (as Table 8).

Ice cream samples were made in an ice cream machine (Uğur Cooling Inc., Aydın, Turkey) and hardened in a deep freezer at −20 °C. All analyses in the study were initiated in duplicate.

#### 4.2.3. Physicochemical Analysis

##### The Physicochemical Analysis of Milk, Cream, Sunflower Oil, and Oleogel Used in Ice Cream Making

Dry matter, ash, fat and oil content and specific weights of milk, cream, and oleogel were measured according to these methods [33]. Specific gravity of milk samples was determined by pycnometer. Dry matter analysis results were obtained by drying the samples at 105 °C in a drying cabinet and ash results by burning the samples in a muffle furnace at 550 °C. The fat content of milk and cream samples was determined the Gerber method [33]. Titratable acidity was determined by a titrimetric method. pH was measured with a pH meter (Seven Compact pH/Ion meter S220; Mettler Toledo, Schwerzenbach, Switzerland) [34]. Milk, cream, and oleogel viscosity were measured at 4 °C using a viscometer (Model DV-II; Brookfield Engineering Labs, Inc., Stoughton, USA) at 20 and 50 rpm [35]. The amounts of total oil in sunflower oil and oleogel were measured using the Soxhlet extractor model PBI (Buchi, Italy) according to AOAC (2000) Method No 30-25 [36]. It is based on the principle of extracting the oil in the sample with a suitable solvent using a soxelet (soxhelet) extraction device. In this way, the amount of oil obtained from the sample is the entire substance extracted under the conditions specified in the method and is expressed as%. 

##### Physicochemical Analysis of Ice Cream Samples

Dry matter, ash, fat and oil content, and specific gravity were determined according to the methods given by the researchers [33]. Titratable acidity was determined by the titrimetric method and pH was analyzed with a pH meter (Seven Compact pH/Ion meter S220; Mettler Toledo, Schwerzenbach, Switzerland) [34].Color determination in ice cream varieties was performed using a chromameter device (CR-300, Konica Minolta, Osaka, Japan) using a D65/10 standard light source, which corresponds to the daylight reading value according to the CIE system. L*, a*, and b* values of the samples were recorded in the reading performed with the device. According to this, the L* value is brightness in daylight (0: black, 100: white), the a* value is green-red (−80 to 0: green, 0 to +50: gray, +50 to +100: red), The b* value represents blue-yellow (from −50 to 0: blue, from 0 to +50: yellow) [37]. Viscosity analyses of the mixes were conducted at 20 and 50 rpm. while the others (sunflower oil, cream, and oleogel) were measured at only 50 rpm using a Brookfield Viscometer (Model DV- II, Brookfield Engineering Laboratories, Stoughton, MA, USA)and cap No: 5. The spindles of viscometers were dipped into samples (ice cream mixes, cream, sunflower oil, and oleogel) at 4 °C. The viscosity values of the samples were determined over 30 s at +4 °C. The shear velocity results of the viscometer were read as centipoise (cP) and these values were calculated in Pa.s [37]. Additionally, overrun was determined using the method proposed by Jimenez-Florez et al. [38]; first dripping and complete melting times were detected using the method described by Cotrell et al. [39].

A technique based on the process was used to calculate the fat destabilization index in ice cream and the melted ice cream samples. For this, ice cream mix (1 g) was diluted 500 times with distilled, deionized water. The samples were then centrifuged for 5 min at 1000 rpm. Absorbance was measured 10 min later at 540 nm on spectrophotometer (Shimadzu UV 3100, Kyoto, Japan). Fat destabilization was calculated as (A mix− A ice cream) × 100/A mix [16].

#### 4.2.4. Fatty Acid Analysis of Ice Cream Samples

The basis of the fatty acid analysis is to determine the methylated fatty acids esters given to the gas chromatography according to the differences in the retention times and to determine their ratio as% according to the obtained peaks. Fatty Acid analyses of ice cream samples were made using the methods proposed by Sukhija and Palmquist [40].

##### Lipid Extraction: Removing Fat and Oil from Ice Cream Sample

Lipid extraction was performed for free fatty acids (FFA) using the modified procedures by Deeth et al. [41]. Two grams of frozen sample were weighed into a 100 mL beaker, then 8 mL of methanol and 18 mL of chloroform were added and homogenized for 30 s. After homogenization, 9 mL of chloroform was added to the beaker and homogenized for another 30 s. Then 9 mL of zinc acetate (0.115 g of zinc acetate/5 mL of deionized water) was added and homogenized for 30 s. The mixtures were then poured into a 125 mL separatory funnel and refrigerated until the two layers were separated. Once two layers were visible, the fat layer was transferred into a capped glass tube and stored in a freezer (−18 °C) until fatty acid analysis [41].

##### Preparation of Fatty Acid Methyl Esters

Fatty acids methyl esters (FAME) were performed according to the (AOAC 996.01) Satchithanandam et al. [42] procedure with some modifications. First, 4 mL of 0.5 N methanolic sodium hydroxide was added to 0.1 g of the extracted fat in a vial. The vial was then connected to a water-cooled condenser and its contents refluxed for 10 min. After refluxing, 5 mL of BF3-methanol reagent (Supleco Inc., Bellafonate, PA, USA) was added to the reaction flask through the concentrator and the contents of the flask were refluxed for another minute. After the vials were cooled, saturated NaCl solution was added to the bottle to float the pentane solution containing the methyl esters on the neck of the vial. Finally, 5 to 7 mL of pentane solution containing methyl esters was transferred to a test tube and a small amount of anhydrous sodium sulfate was added to dry the pentane solution.

##### Analysis of Fatty Acid Methyl Esters in Gas Chromatography (GC)

The fatty acid methyl ester (FAME) composition was analyzed using a Thermo Electronic (Austin, TX, USA) gas chromatograph-MS (Model TRACE GC Ultra) equipped with (Model AS-3000-Thermo Electronic Company) autosampler using a silica capillary column (0.25 mm id × 0.25 µm film thickness x 60-m; SP-2380 Supelco. Inc. Bellefonte, PA, USA) and a flame ionization detector (FID).The injection temperature was programmed between 10 °C and 220 °C at 2 °C/min. Helium was used as the carrier gas with a flow rate of 1.6 mL/min and a partition ratio of 30: 1. Analysis of the fatty acids profile was determined as fatty acid methyl esters. Next, 300-μL of thawed and thoroughly mixed sample was taken in an 11-mL screw-capped test tube. Samples dissolved in 3 mL of isooctane and 2 mL of 1.5 N sodium methoxide were then vortexed at a higher rate for exactly 3 min and allowed to separate for 5 min, and the supernatant was injected into the gas chromatograph. The injection split ratio was 1:80. A Lab Solution computer program was used to control the GC/FID system, and the standard FAME mix (Supelco 37 component FAME mix, Supelco^®^, Bellefonte, PA, USA) was used as a standard. FAME peaks were identified by comparing results against the retention times and chain lengths specified in the FAME standard [41].

#### 4.2.5. Optical Microscopy

The size and distribution of bubbles in the ice creams were observed and analyzed using a light microscope with integrated camera. After the samples were placed on a slide, a coverslip was used to cover them and observed with an optical microscope (Zeiss Axio Scope A1, Carl Zeiss Co., Ltd., Jena, Germany) at 20 × magnification. Images were transferred to a computer using a digital camera (Sony CCD Color Digital Video C-Mount Microscope Camera, Tokyo, Japan) and analyzed with the Image-Pro Plus 6.0 (Media Cybernetics Inc, Bethesda, Rockville, MD, USA) software program [43].

#### 4.2.6. Sensory Assessment

Ice cream samples were placed in special 100 mL odor-free containers with glass lids and presented to the panelists in a randomly coded manner at regular intervals. While the panelists were performing sensory analysis, water was placed in 250 mL glass bottle containers to clean their mouths before moving on to the next sample. Sensory evaluations were made by considering color and appearance, texture and consistency, taste and smell, sweetness and gummy structure and general acceptability.Sensory evaluation was performed in a spaced seating arrangement in a room at an appropriate temperature (25 °C). Ice cream samples (–10 °C) were presented to the panelists. Samples were evaluated by panelists of 10 experts (academician, doctoral, graduate and undergraduate students) from the Department of Food Engineering using scoring tables from 1 to 9.Each panelist was an experienced individual trained and informed about sensory analysis methodology [44].

#### 4.2.7. Statistical Analysis

The trial was set up as 2 (addition of oleogel and cream) × 2 (addition of stabilizer) × 3 (addition of emulsifier) according to a 2 × 2 × 3 fully random factorial trial plan. The experiment was set up according to a 2 × 2 × 3 full random factorial experiment plan, as 2 (addition of oleogel and cream) × 2 (addition of stabilizer) × 3 (addition of emulsifier). The experiment was carried out in three replications. Two-way analysis of variance (TWO-way ANOVA) was used for the determination the differences between the ice cream samples and the effects of storage. Accordingly, the statistical analysis software program SPSS version 15.0 (SPSS Inc., Chicago, IL, USA) was used. The significant data as a result of analysis of variance (ANOVA) were tested according to the Duncan multiple comparison test at *p* < 0.01 level.

## Figures and Tables

**Table 1:** Some physicochemical properties of milk, cream, sunflower oil, and oleogel.

Samples	Dry Matter (%)	Fat and Oil Ratio (%)	Ash (%)	Sspecific Gravity	Acidity (%)	pH	Viscosity (Pa.s.) (50 rpm)
Milk (all fat)	12.70	3.30	0.75	1.028	0.19	6.47	-
Cream	55.50	66.80	0.55	-	1.19	6.51	1.32
Milk (no fat)	10.03	0.10	0.72	1.036	0.16	6.13	-
Sunflower oil (refined)	-	99.75	-	0.918	0.51	6.81	1.15
Oleogel	-	95.25	-	-	0.43	6.34	1.61

**Table 2:** The results of physicochemical analysis of the mix and ice cream samples.

Sample	Dry Matter (%)	Milk Fat/ Oil Ratios (%)	Ash (%)	pH	Acidity (%)	Overrun (%)	Mix Viscosity (Pa.s.)	
							(20 rpm)	(50 rpm)
A	31.37 ± 1.1 ^a^	5.05 ± 0.1 ^e^ (MF)	0.96 ± 0.2 ^a^	6.71 ± 0.4 ^a^	0.14 ± 0.01 ^c^	34.84 ± 5.1 ^b^	12.68 ± 1.4 ^d^	8.07 ± 0.8 ^cd^
B	29.87 ± 1.8 ^b^	5.10± 0.4 ^e^ (MF)	0.83 ± 0.1 ^ab^	6.17 ± 0.3 ^b^	0.18 ± 0.02 ^a^	42.10 ± 8.2 ^a^	14.71 ± 2.1 ^c^	9.21 ± 1.5 ^c^
C	32.35 ± 0. 7 ^a^	4.65 ± 0.1 ^f^ (MF)	0.75 ± 0.01 ^c^	6.66 ± 0.08 ^a^	0.15 ± 0.01 ^b^	30.67 ± 3.4 ^bc^	11.72 ± 8.9 ^d^	7.19 ± 4.1 ^d^
D	31.50 ± 1.9 ^a^	5.10 ± 0.1 ^e^ (MF)	0.81 ± 0.03 ^b^	6.64 ± 0.6 ^a^	0.14 ± 0.10 ^c^	26.03 ± 4.5 ^c^	24.32 ± 2.5 ^a^	13.03 ± 1.4 ^a^
E	28.98 ± 2.9 ^c^	5.07 ± 0.1 ^e^ (MF)	0.80 ± 0.1 ^b^	5.98 ± 0.4 ^c^	0.16 ± 0.01 ^b^	32.49 ± 1.9 ^b^	20.69 ± 4.0 ^b^	11.08 ± 0.1 ^ab^
F	33.13 ± 2.4 ^a^	5.00± 0.8 ^e^ (MF)	0.86 ± 0.9 ^a^	6.70 ± 0.5 ^a^	0.14 ± 0.04 ^c^	29.83 ± 12.4 ^b c^	26.33 ± 10.3 ^a^	11.26 ± 3.0 ^ab^
G	29.89 ± 0.1 ^b^	6.28 ± 0.2 ^c^ (oil)	0.79 ± 0.05 ^b^	6.02 ± 0.1 ^c^	0.18 ± 0.0 ^a^	12.35 ± 3.6 ^e^	11.82 ± 1.3 ^d^	7.00 ± 0.3 ^d^
H	29.82 ± 0.5 ^b^	6.55 ± 0.2 ^a^ (oil)	0.90 ± 0.1 ^a^	6.25 ± 0.1 ^b^	0.14 ± 0.01 ^c^	17.56 ± 7.9 ^de^	10.06 ± 6.0 ^d^	6.58 ± 2.0 ^d^
I	30.65 ± 0.6 ^b^	6.30 ± 0.3 ^c^ (oil)	0.82 ± 0.6 ^ab^	6.55 ± 0.1 ^ab^	0.14 ± 0.03 ^c^	6.50 ± 17.1 ^f^	18.61 ± 2.7 ^b^	9.50 ± 1.7 ^b^
J	31.58 ± 0.3 ^a^	6.15 ± 0.07 ^d^ (oil)	0.97 ± 0.2 ^a^	6.48 ± 0.5 ^ab^	0.13 ± 0.02 ^cd^	30.87 ± 14.0 ^bc^	22.40 ± 3.4 ^ab^	11.96 ± 1.2 ^ab^
K	32.20 ± 0.7 ^a^	6.50 ± 0.2 ^a^ (oil)	0.72 ± 0.01 ^d^	5.91 ± 0.3 ^c^	0.16 ± 0.03 ^b^	11.18 ± 7.8 ^e^	17.69 ^b^ ± 7.28	10.42 ± 3.1 ^b^
L	31.26 ± 0.01 ^a^	6.45 ± 0.01 ^b^ (oil)	0.80 ± 0.01 ^b^	6.51 ± 0.01 ^ab^	0.12 ± 0.01 ^d^	22.35 ± 0.01 ^d^	28.00 ^a^ ± 0.01	14.70 ± 0.01 ^a^

Note: Different letters indicate that the averages are different from each other.

**Table 3:** Melting properties and fat destabilization of ice cream samples.

Samples	First Drop. Time (min.)	Complete Melting Time (min.)	Melting Ratio (%)	Fat Destabilization (%)
A	12.50 ± 3.5 ^g^	64.50 ± 2.1 ^e^	96.05 ± 1.9 ^a^	27.30 ± 1.5 ^b^
B	19.00 ± 4.2 ^ef^	64.00 ± 4.2 ^e^	90.12 ± 0.3 ^b^	21.15 ± 0.9 ^e^
C	17.00 ± 0.01 ^f^	63.50 ± 1.4 ^e^	95.78 ± 3.1 ^a^	37.50 ± 1.4 ^a^
D	43.50 ± 2.1 ^bc^	66.00 ± 1.4 ^e^	81.96 ± 7.4 ^c^	31.11 ± 0.9 ^b^
E	32.50 ± 3.5 ^d^	77.00 ± 4.2 ^c^	79.68 ± 10.5 ^c^	23.32 ± 2.2 ^d^
F	35.00 ± 5.7 ^c^	79.50 ± 2.1 ^c^	66.96 ± 0.3 ^d^	28.90 ± 0.6 ^b^
G	26.50 ± 0.7 ^e^	94.50 ± 2.1 ^a^	66.69 ± 1.4 ^d^	22.59 ± 1.4 ^d^
H	16.50 ± 0.7 ^f^	72.00 ± 2.8 ^d^	95.74 ± 5.2 ^a^	24.79 ± 0.9 ^c^
I	31.00 ± 0.01 ^d^	93.50 ± 2.1 ^a^	66.81 ± 1.6 ^d^	20.84 ± 1.1 ^e^
J	37.50 ± 0.7 ^c^	84.50 ± 3.5 ^b^	68.02 ± 5.6 ^d^	18.71 ± 0.2 ^f^
K	49.00 ± 1.4 ^b^	89.00 ± 1.4 ^a^	47.71 ± 1.4 ^e^	19.36 ± 0.1 ^f^
L	60.50 ± 0.7 ^a^	88.50 ± 0.7 ^a^	82.00 ± 0.3 ^c^	21.20 ± 0.5 ^e^

Note: Different letters indicate that the averages are different from each other.

**Table 4:** The color values of various ice cream samples.

Samples	L* Color	a* Color	b* Color
A	85.47 ± 4.1 ^a^	−2.88 ± 0.3 ^c^	9.56 ± 1.05 ^a^
B	80.88 ± 0.8 ^c^	−2.36 ± 0.04 ^c^	8.06 ± 0.6 ^bc^
C	80.83 ± 3.9 ^c^	−2.72 ± 0.2 ^c^	8.70 ± 0.7 ^ab^
D	81.33 ± 0.9 ^c^	−2.65 ± 0.1 ^c^	8.76 ± 1.1 ^ab^
E	86.76 ± 5.3 ^a^	−2.08 ± 0.02 ^b^	7.50 ± 0.04 ^c^
F	86.05 ± 0.9 ^a^	−3.08 ± 0.09 ^c^	8.22 ± 0.01 ^b^
G	83.61 ± 6.4 ^b^	−3.13 ± 0.2 ^c^	8.33 ± 0.2 ^b^
H	83.14 ± 5.1 ^b^	−3.02 ± 0.2 ^c^	7.96 ± 0.4 ^bc^
I	80.66 ± 7.1 ^c^	−2.77 ± 0.2 ^c^	7.03 ± 0.3 ^d^
J	81.16 ± 5.2 ^c^	−3.34 ± 0.2 ^d^	8.14 ± 0.3 ^b^
K	87.15 ± 1.6 ^a^	−3.30 ± 0.1 ^d^	8.64 ± 0.1 ^ab^
L	83.94 ± 2.7 ^b^	−1.84 ± 0.04 ^a^	7.00 ± 0.6 ^d^

Note: Different letters indicate that the averages are different from each other.

**Table 5:** The fatty acids composition of ice cream samples.

Samples	C4:0	C6:0	C14:0	C16:0	C18:0	C18:1	C18:2	C18:3
A	2.25 ± 0.10 ^a^	1.67 ± 0.03 ^a^	12.32 ± 0.03 ^a^	35.85 ± 0.10 ^a^	10.33 ± 0.04 ^c^	22.25 ± 0.10 ^a^	1.51 ± 0.01 ^bc^	nd ^b^
B	2.25 ± 0.10 ^a^	1.55 ± 0.10 ^a^	10.40 ± 0.07 ^b^	35.23 ± 0.04 ^a^	13.03 ± 0.04 ^a^	21.46 ± 0.10 ^b^	3.03 ± 0.04 ^b^	nd ^b^
C	2.01 ± 0.01 ^a^	1.50 ± 0.10 ^a^	12.11 ± 0.01 ^a^	34.77 ± 0.10 ^a^	11.41 ± 0.01 ^b^	22.50 ± 0.10 ^a^	1.03 ± 0.04 ^bc^	nd ^b^
D	2.16 ± 0.10 ^a^	1.50 ± 0.10 ^a^	10.29 ± 0.10 ^b^	34.34 ± 0.10 ^a^	12.19 ± 0.10 ^b^	22.19 ± 0.10 ^a^	3.91 ± 0.01 ^b^	nd ^b^
E	2.08 ± 0.03 ^a^	1.50 ± 0.10 ^a^	12.47 ± 0.10 ^a^	36.09 ± 0.00 ^a^	10.61 ± 0.01 ^c^	22.11 ± 0.00 ^a^	2.71 ± 0.01 ^b^	nd ^b^
F	1.77 ± 0.10 ^b^	1.35 ± 0.10 ^b^	11.63 ± 0.04 ^a^	35.12 ± 0.10 ^a^	10.86 ± 0.10 ^c^	22.75 ± 0.10 ^a^	2.84 ± 0.10 ^b^	nd ^b^
G	nd ^c^	nd ^c^	0.180 ± 0.04 ^c^	6.480 ± 0.10 ^c^	4.110 ± 0.01 ^d^	23.37 ± 0.10 ^a^	65.75 ± 0.10 ^a^	0.10 ± 0.01 ^a^
H	nd ^c^	nd ^c^	0.230 ± 0.04 ^c^	8.300 ± 0.03 ^b^	5.040 ± 0.10 ^d^	22.05 ± 0.10 ^a^	64.04 ± 0.10 ^a^	0.12 ± 0.03 ^a^
I	nd ^c^	nd ^c^	0.250 ± 0.10 ^c^	6.620 ± 0.04 ^c^	4.210 ± 0.01 ^d^	23.68 ± 0.10 ^a^	64.23 ± 0.04 ^a^	0.14 ± 0.03 ^a^
J	nd ^c^	nd ^c^	0.210 ± 0.00 ^c^	7.830 ± 0.10 ^b^	4.840 ± 0.10 ^d^	22.26 ± 0.10 ^a^	64.52 ± 0.03 ^a^	0.12 ± 0.03 ^a^
K	nd ^c^	nd ^c^	0.220 ± 0.03 ^c^	6.440 ± 0.10 ^c^	4.120 ± 0.03 ^d^	22.96 ± 0.10 ^a^	65.80 ± 0.10 ^a^	0.13 ± 0.03 ^a^
L	nd ^c^	nd ^c^	0.170 ± 0.03 ^c^	6.630 ± 0.04 ^c^	4.160 ± 0.10 ^d^	23.47 ± 0.00 ^a^	65.19 ± 0.01 ^a^	0.14 ± 0.03 ^a^

Note: Different letters indicate that the averages are different from each other.

**Table 6:** The correlations test results of ice cream samples.

Samples	First Drop Time	Complete Melting Time	Melting Ratio	Dry Matter	Fat or Oleogel	pH	Overrun	L*	a*	b*	Fat Desatabilization	Viscosity (20 rpm)	Viscosity (50 rpm)	C4:0	C6:0	C14:0	C16:0	C18:1	C18:2	C18:3
First Drop.Time	1.00	0.534 **	−0.580 **	0.26	0.39	−0.09	−0.32	0.09	0.22	−0.37	−0.35	0.809 **	0.856 **	−0.38	−0.439 *	−0.39	−0.39	0.37	0.38	0.419 *
Co.Melt.Time	0.53 **	1.00	−0.752 **	−0.05	0.734 **	−0.32	−0.743 **	−0.16	−0.17	−0.467 *	−0.607 **	0.27	0.20	−.803 **	−0.821 **	−0.754 **	−0.767 **	0.824 **	0.780 **	0.770 **
Mel.Ratio	−0.58 **	−0.752 **	1.00	−0.23	−0.439 *	0.39	0.549 **	0.00	0.408 *	0.15	0.513 *	−0.36	−0.30	0.510 *	0.625 **	0.472 *	0.553 **	−0.468 *	−0.482 *	−0.444 *
Dry matter	0.26	−0.05	−0.23	1.00	−0.20	0.569 **	0.05	0.15	−0.37	0.36	0.40	0.34	0.25	0.09	−0.12	0.14	−0.13	0.15	−0.14	−0.07
Fat/oleogel	0.39	0.734 **	−0.439 *	−0.20	1.00	−0.37	−0.751 **	−0.12	−0.21	−0.39	−0.702 **	0.00	0.05	−0.947 **	−0.809 **	−0.965 **	−0.775 **	0.554 **	0.967 **	0.951 **
pH	−0.09	−0.32	0.39	0.569 **	−0.37	1.00	0.25	−0.25	0.02	0.14	0.550 **	0.28	0.18	0.31	0.19	0.33	0.19	0.02	−0.34	−0.23
Overrun	−0.32	−0.743 **	0.549 **	0.05	−0.751 **	0.25	1.00	0.26	0.31	0.27	0.30	0.07	0.11	0.787 **	0.698 **	0.757 **	0.757 **	−0.715 **	−0.768 **	−0.748 **
L*	0.09	−0.16	0.00	0.15	−0.12	−0.25	0.26	1.00	−0.03	0.32	0.07	0.06	0.08	0.17	0.07	0.21	0.04	−0.18	−0.18	−0.22
a*	0.22	−0.17	0.408 *	−0.37	−0.21	0.02	0.31	−0.03	1.00	−0.490 *	0.01	0.30	0.40	0.32	0.38	0.28	0.30	−0.06	−0.29	−0.20
b*	−0.37	−0.467 *	0.15	0.36	−0.39	0.14	0.27	0.32	−0.490 *	1.00	0.412 *	−0.35	−0.32	0.39	0.409 *	0.38	0.38	−0.31	−0.38	−0.444 *
Fat Destab.	−0.35	−0.60 **	0.513 *	0.40	−0.702 **	0.550 **	0.30	0.07	0.01	0.412 *	1.00	−0.16	−0.24	0.610 **	0.528 **	0.656 **	0.431 *	−0.26	−0.655 **	−0.631 **
Visc.(20)	0.81 **	0.27	−0.36	0.34	0.00	0.28	0.07	0.06	0.30	−0.35	−0.16	1.00	0.953 **	−0.01	−0.23	0.00	−0.15	0.16	−0.01	0.05
Visc.(50)	0.85 **	0.20	−0.30	0.25	0.05	0.18	0.11	0.08	0.40	−0.32	−0.24	0.953 **	1.00	−0.01	−0.12	−0.04	−0.03	0.06	0.02	0.09
C4:0	−0.38	−0.803 **	0.510 *	0.09	−0.947 **	0.31	0.787 **	0.17	0.32	0.39	0.610 **	−0.01	−0.01	1.00	0.890 **	0.984 **	0.823 **	−0.627 **	−0.992 **	−0.954 **
C6:0	−0.44 *	−0.821 **	0.625 **	−0.12	−0.809 **	0.19	0.698 **	0.07	0.38	0.409 *	0.528 **	−0.23	−0.12	0.890 **	1.00	0.832 **	0.949 **	−0.646 **	−0.843 **	−0.818 **
C14:0	−0.39	−0.754 **	0.472 *	0.14	−0.965 **	0.33	0.757 **	0.21	0.28	0.38	0.656 **	0.00	−0.04	0.984 **	0.832 **	1.00	0.762 **	−0.558 **	−0.995 **	−0.955 **
C16:0	−0.39	−0.767 **	0.553 **	−0.13	−0.775 **	0.19	0.757 **	0.04	0.30	0.38	0.431 *	−0.15	−0.03	0.823 **	0.949 **	0.762 **	1.00	−0.703 **	−0.773 **	−0.760 **
C18:1	0.37	0.824 **	−0.468 *	0.15	0.554 **	0.02	−0.715 **	−0.18	−0.06	−0.31	−0.26	0.16	0.06	−0.627 **	−0.646 **	−0.558 **	−0.703 **	1.00	0.594 **	0.632 **
C18:2	0.38	0.780 **	0.482 *	−0.14	0.967 **	−0.34	−0.768 **	−0.18	−0.29	−0.38	−0.655 **	−0.01	0.02	−0.992 **	−0.843 **	−0.995 **	−0.773 **	0.594 **	1.00	0.961 **
C18:3	0.42 *	0.770 **	−0.444 *	−0.07	0.951 **	−0.23	−0.748 **	−0.22	−0.20	−0.444 *	−0.631 **	0.05	0.09	−0.954 **	−0.818 **	−0.955 **	−0.760 **	0.632 **	0.961 **	1.00

Variables of Significance (*: *p* < 0.05; **: *p* < 0.01).

**Table 7:** The results of sensory analysis of ice cream samples.

Samples	Color Appearance	Texture Consistency	Gummy	Taste Smell	Sweetness	General Acceptability
A	8.50	8.00	8.00	8.00	7.50	8.00
B	9.00	8.00	7.00	7.50	7.50	7.00
C	8.50	8.50	8.00	9.00	8.50	9.00
D	8.00	8.00	7.00	7.50	6.50	6.50
E	8.00	8.00	7.00	7.00	7.00	7.00
F	9.00	8.00	8.00	7.50	7.50	7.50
G	8.00	7.00	7.00	5.00	6.00	5.00
H	7.50	7.50	7.00	7.00	7.50	7.00
I	7.50	7.50	7.50	6.50	7.00	6.50
J	8.50	8.00	8.00	7.50	7.50	7.50
K	8.50	7.50	6.50	7.00	7.50	7.00
L	9.00	8.00	8.00	7.00	7.50	6.50

Note: Items were scored as a maximum of 9 by panelists.

**Table 8:** The content of components used in ice cream mix formulations.

Samples	Fat or Oil Sources	Stabilizers	Emulsifiers	Milk	Milk Powder	Raw Cream	Sucrose
A	milk fat	Salep	NE	fat	+	+	+
B	milk fat	Salep	MG	fat	+	+	+
C	milk fat	Salep	PG	fat	+	+	+
D	milk fat	KG	NE	fat	+	+	+
E	milk fat	KG	MG	fat	+	+	+
F	milk fat	KG	PG	fat	+	+	+
G	oleogel	Salep	NE	non fat	+	_	+
H	Oleogel	Salep	MG	non fat	+	_	+
I	Oleogel	Salep	PG	non fat	+	_	+
J	Oleogel	KG	NE	non fat	+	_	+
K	Oleogel	KG	MG	non fat	+	_	+
L	Oleogel	KG	PG	non fat	+	_	+

## Data Availability

The author declare that all the data supporting the findings of this study are available within the article.

## Abbreviations

(MG) Monoglyceride; (PG) Palsgaard; (NE) Emulsifier-free; (PG) Palsgaard; (BW) beeswax; (CDW) candelilla wax; (CBW) carnauba wax; (CMC) carboxy methyl cellulose; (GG) guar gum; (RBW) rice bran wax; (KG) konjac gum; (XG) Xanthan gum; (FFA) free fatty acids; (LBG) locust bean gum; (FAME) Fatty acids methyl esters; (GC) chromatography flame; (FID) ionization detector; (MF) Milk Fat; (ANOVA) analysis of variance; (WHO) World Health Organization; (nd) None detected; (NE) none emulsifier.
